# Epidemiology and Molecular Characterization of Rotavirus A in Fruit Bats in Bangladesh

**DOI:** 10.1007/s10393-020-01488-7

**Published:** 2020-09-02

**Authors:** Ariful Islam, Mohammad Enayet Hossain, Melinda K. Rostal, Jinnat Ferdous, Ausraful Islam, Rashedul Hasan, Mojnu Miah, Mustafizur Rahman, Mohammed Ziaur Rahman, Peter Daszak, Jonathan H. Epstein

**Affiliations:** 1 EcoHealth Alliance, 460 West 34th Street, Suite 17, New York, NY 10001 USA; 2 Centre for Integrative Ecology, School of Life and Environmental Sciences, Deakin University, Geelong, VIC Australia; 3 International Centre for Diarrheal Diseases Research, Bangladesh (icddr,b), Dhaka, Bangladesh; 4 Institute of Epidemiology, Disease Control and Research (IEDCR), Mohakhali, Dhaka, 1212 Bangladesh

**Keywords:** Rotavirus A, *Pteropus medius*, *Rousettus leschenaultii*, *Taphozous melanopogon*, G1, G8

## Abstract

Rotavirus A (RVA) is the primary cause of acute dehydrating diarrhea in human and numerous animal species. Animal-to-human interspecies transmission is one of the evolutionary mechanisms driving rotavirus strain diversity in humans. We screened fresh feces from 416 bats (201 *Pteropus medius*, 165 *Rousettus leschenaultii* and 50 *Taphozous melanopogon*) for RVA using rRT-PCR. We detected a prevalence of 7% (95% CI 3.5–10.8) and 2% (95% CI 0.4–5.2) in *P. medius* and *R. leschenaultii*, respectively. We did not detect RVA in the insectivorous bat (*T. melanopogon*). We identified RVA strains similar to the human strains of G1 and G8 based on sequence-based genotyping, which underscores the importance of including wildlife species in surveillance for zoonotic pathogens to understand pathogen transmission and evolution better.

Globally, group A rotavirus (RVAs) is the primary cause of acute dehydrating diarrhea in people, especially in children (Karampatsas et al. 2018). It also causes disease in a number of animal species (Dhama et al. 2009). RVAs are estimated to cause 215,000 deaths per year globally in children (< 5 years) (Tate et al. 2016). There is a significant amount of RVA strain diversity, and a variety of human strains share genetic and antigenic features with animal origin RVA strains (Luchs and Timenetsky 2014). Interspecies transmission is an important mechanism of RVA evolution and can contribute to the diversity of human RVA strains (Do et al. 2016). However, heterologous RVA infections (such as a bovine RVA isolate infecting other species) have typically resulted in lower viral titers and an absence of severe diarrhea (Sieg et al. 2015) in people.

Approximately 60% of emerging infectious diseases are zoonotic, and the majority of those (72%) originate in wildlife (Jones et al. 2008). Bats are believed to be the reservoir of RVA (He et al. 2017). RVA was found in two straw-colored fruit bats (*Eidolon helvum*) and an Egyptian fruit bat (*Rousettus* *aegyptiacus*) in Zambia (Sasaki et al. 2018); three *E. helvum*, a lesser mouse-tailed bat (*Rhinopoma hardwickii)* and an Egyptian tomb bat (*Taphozous perforatus*) in Saudi Arabia (Mishra et al. 2019); and in 38 (out of 305; 9.2%) Brazilian bats (Asano et al. 2016). Other studies have identified novel strains of RVA genotype from different species of bats, of which some were closely related to human RVAs (Kim et al. 2016; Yinda et al. 2016).

The general prevalence of rotavirus in human and animals like cattle, buffalo, goat and chicken were reported to be 23.8% and 12–43%, respectively, in Bangladesh (Samad 2013). A study in Bangladesh identified a human RVA strain in which the VP3 gene sequence was more closely related to an RVA strain previously found in goats (Ghosh et al. 2011), suggesting possible interspecies RVA transmission. Kumar and Malik (Kumar and Malik 2018) also indicated that reassortment facilitated the infection of calves with an RVA strain that had a G1 gene related to a human RVA, the evolution of an emerging pathogen, which could be a threat to public health (Ghosh et al. 2011). Though rotaviral diarrhea in humans is common in Bangladesh (Pecenka et al. 2017), there are few data regarding RVA and its genetic diversity and geographic distribution within bats of Bangladesh. The current study was conducted to detect RVA within several species of bats and assess their similarity to human strains.

The study was conducted under ethical approval from the International Centre for Diarrheal Disease Research, Bangladesh (icddr,b; protocol: 2008-074), and University of California, Davis (protocol: 16048). We collected noninvasive fecal samples (*n* = 416) during a four-year period (2011–14) from northwest Bangladesh. We collected 165 fecal samples from small fruit bats (*Rousettus leschenaultii)* in Dupadanga and Nolia, Rajbari district; 201 fecal samples from large fruit bat species (Indian flying fox; *Pteropus medius)* in Dhaka, Faridpur, Manikganj and Tangail districts; and 50 fecal samples from the insectivorous black-bearded tomb bat (*Taphozous melanopogon)* in Orakandi and Ramnagar, Rajbari district (Fig. 1).

**Figure 1:**
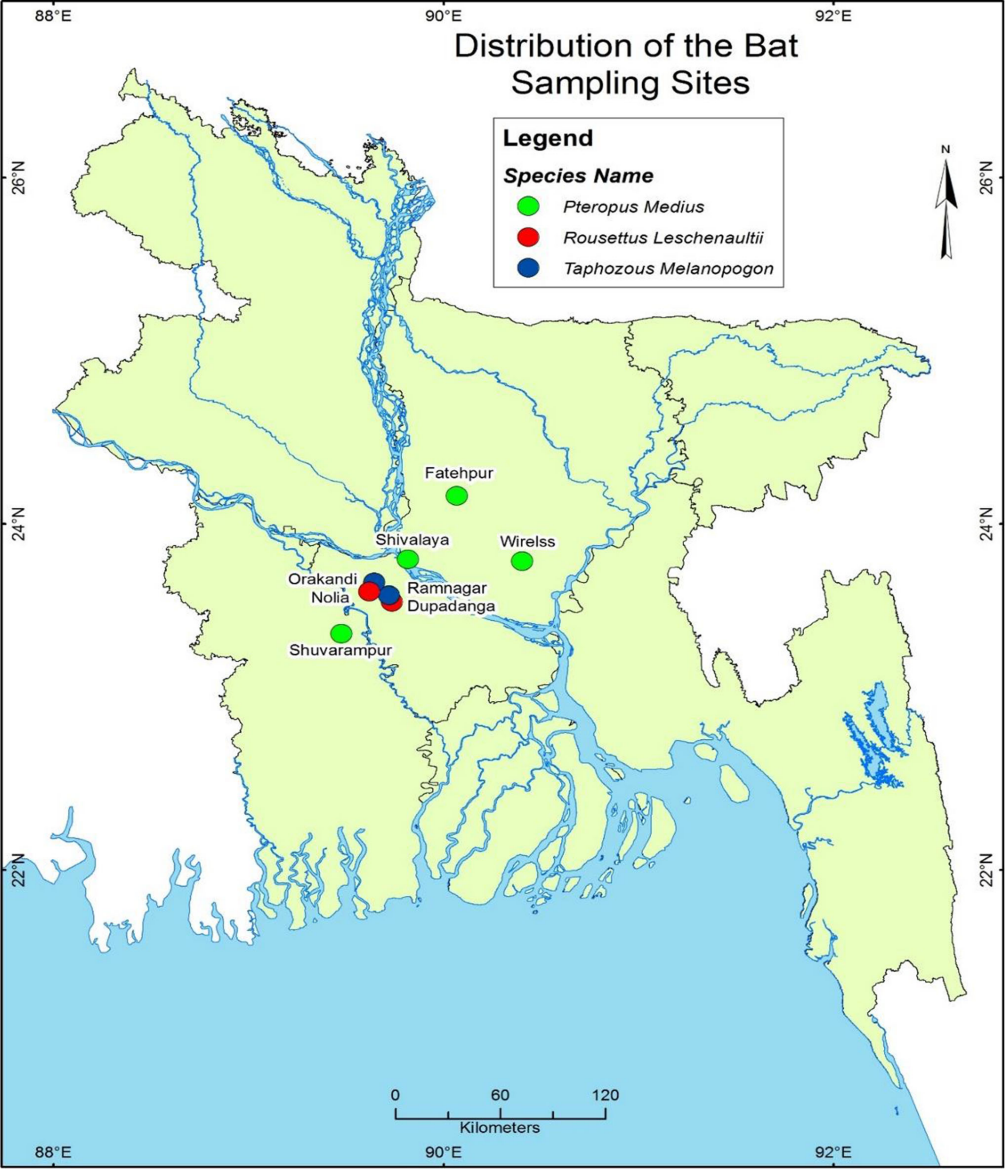
Distribution of the bat sampling locations from 2011 to 2014.

We collected fecal samples between 3 and 6 am following the procedure described by Swift et al. 2018. The tubes containing sample and nuclease lysis buffer were stored in a liquid nitrogen Dewar (Princeton Cryogenics, NJ, USA) in the field until being transferred to a − 80°C freezer at laboratory. We tested fecal swabs for RVA RNA by real-time reverse transcription polymerase chain reaction (rRT-PCR) using NSP3-specific primers and probes using the AgPath-ID™ One-Step RT-PCR system (Ambion Inc. Austin, USA) according to Jothikumar et al. (2009). To identify the G and P genotype, we performed conventional reverse transcription polymerase chain reaction (RT-PCR) to amplify the VP7 and VP4 gene fragments using consensus primer pairs Beg9/End9 and Con2/Con3, respectively (Table 1), as described elsewhere (Islam et al. 2019; Rahman et al. 2007). We carried out RT-PCR using the QIAGEN^®^ One-Step RT-PCR Kit (QIAGEN, Germany) according to the manufacturer’s instructions. Nucleotide sequencing was carried out using the di-deoxynucleotide chain termination method with the ABI PRISM BigDye Terminator Cycle Sequencing Reaction kit v3.1 (Life Technologies Corp., Carlsbad, CA 92008 USA) in an automated genetic analyzer (ABI 3500xL).The electropherogram files of the nucleotide sequences were examined and edited using Chromas 2.23 (Technelysium).

**Table 1:** Oligonucleotide primers used in the study for PCR amplification.

Primer	Type	Position	Strand	Sequence (5′–3′)	References
Forward	JVKF	17–39	Plus	CAGTGGTTGATGCTCAAGATGGA	Jothikumar et al. (2009)
Reverse	JVKR	147–123	minus	TCATTGTAATCATATTGAATACCCA	Jothikumar et al. (2009)
Probe	JVKP	96–72	Plus	FAM-ACAACTGCAGCTTCAAAAGAAGWGT-BHQ1	Jothikumar et al. (2009)
Beg9	VP7	1–28	Plus	GGCTTTAAAAGAGAGAATTTCCGTCTGG	Farkas et al. 2008
End9	VP7	1062–1036	Minus	GGTCACATCATACAATTCTAATCTAAG	Farkas et al. (2008)
Con2	VP4	868–887	Minus	ATTTCGGACCATTTATAACC	Farkas et al. (2008)
Con3	VP4	11–32	Plus	TGGCTTCGCCATTTTATAGACA	Farkas et al. (2008)

We submitted the sequences from this study to GenBank under the accession numbers MK674285 and MK674286. Sequence similarity searches were performed using the Basic Local Alignment Search Tool (BLAST) server on the GenBank database. Sequences which had 100% query coverage and 98–99% identity were selected for phylogenetic analysis. Phylogenetic trees were constructed according to the maximum likelihood method using MEGA (Molecular Evolutionary Genetics Analysis) version 6.0 (Tamura et al. 2011). Genotypes were determined by submitting the sequences to online rotavirus genotyping tool RotaC (http://rotac.regatools.be/). We used STATA-13 (StataCorp, 4905, Lakeway Drive, College Station, Texas 77845, USA) software for statistical analysis. We expressed the results as prevalence and their 95% confidence interval (CI).

Seven percent (*n* = 201; 95% CI 3–11) of *P. medius* and 2% (*n* = 165; 95% CI 0–5) of *R. leschenaultii* were positive for RVA. No RVA RNA was detected in *T. melanopogon* (Table 2). 24% (*n* = 55; 95% CI 13–37%) of *P. medius* sampled in Dhaka were found to carry RVA, whereas *P. medius* from all other sites were negative (*n* = 146; 95% CI 0–2.5) (Table 2).

**Table 2:** Rotavirus A RNA detection in the feces of fruit bats of Bangladesh, 2011–2014.

Species	Location	*N*	Positive *n* (%)	95% CI	
*Pteropus medius*	Urban	Dhaka	55	13 (23.6)	13–37
	Rural	Faridpur	11	0	0–28.5
		Manikganj	75	0	0–4.8
		Tangail	60	0	0–5.9
Subtotal			201	13 (6.5)	3.5–10.8
*Rousettus leschenaultii*	Rural	Dupadanga	5	1 (20)	0.5–71
		Nolia, Rajbari	160	2 (1.3)	0.15–4.4
Subtotal			165	3 (1.8)	0.4–5.2
*Taphozous melanopogon*	Rural	Orakandi, Rajbari	30	0	0–11.5
		Ramnagar, Rajbari	20	0	0–16.8
Subtotal			50	–	0–7.1
Total			416	16 (3.8)	2.21–6.2

G1 and G8 genotypes were detected via VP7 genotyping, which was successful for 13% (2/16) of the RNA-positive samples. P genotyping was not successful. Both of the genotyped strains were detected in Dhaka City, Bangladesh. The sequence of the bat G1 and G8 genotypes had high similarity with the human RVA strains, 90% and 97.7–98.7%, respectively. The bat G8 genotype also had high similarity with bovine strains (94–98.7%) from India and porcine strains (84.5%) from South Korea (Fig. 2).

**Figure 2:**
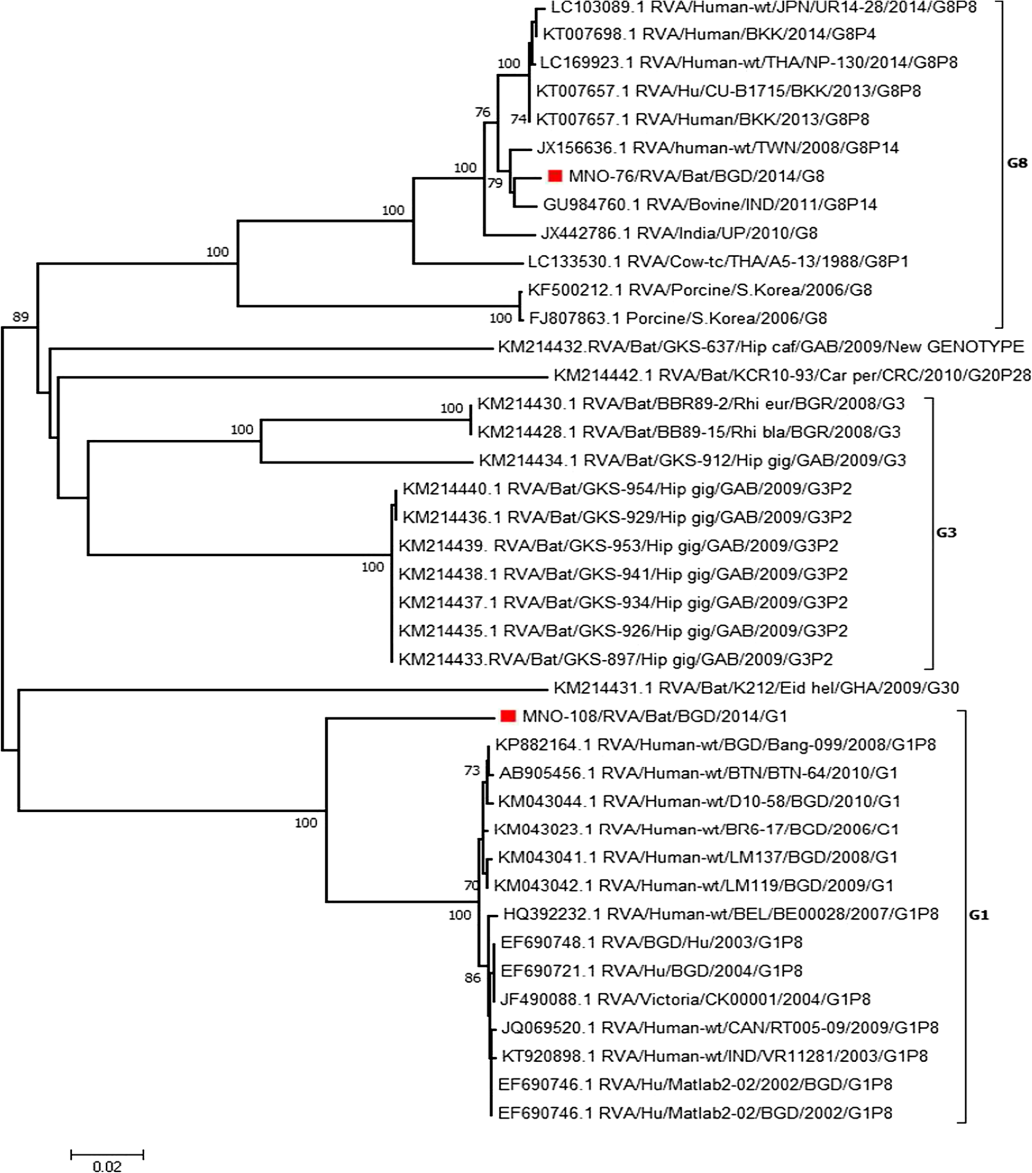
Phylogenetic trees based on partial nucleotide sequences of VP7. The bat RVA strains identified in this study were marked by red square (Color figure online).

To the authors’ knowledge, RVA has not previously been reported in bats from Bangladesh. Our study identified RVA strains in *P. medius* and *R. leschenaultii*. Sasaki et al. (2018) also identified RVA in *Rossettus sp.* (*n* = 1 out of 20), though it was a different species (*R. aegyptiacus*) from Zambia. Our study did not detect RVA RNA in *Taphozous* sp.; however, a previous study identified one *T. perforatus* (*n* = 1/17) carrying RVA in Saudi Arabia (Mishra et al. 2019). RVA was detected at a higher prevalence (9.18%; *n* = 28/305) in bats from Brazil (Asano et al. 2016) and at lower prevalence (0.7%; *n* = 4/457) from China (He et al. 2017) than our study.

The identification of genotypes related to human RVA strains suggests that anthropozoonotic transmission may be occurring between people and bats. While bat-specific RVAs have been identified (Jiang et al. 2004), the genetic sequences that we detected suggest that a reassortment event may have occurred. Habitat destruction and urbanization are hypothesized to drive increasing interactions between people and bat species (Kung et al. 2015). *Pteropus* bats are known to roost in proximity to people, which may require them to share food and water resources (Luskin 2010). Bats have been reported drinking from surface water reservoirs (Stier 2003) and have a habit of immersing their body in the water. In Bangladesh, human and livestock waste is often disposed of in water reservoirs, which can also be contaminated if rain water washes fecal material into the reservoir.

Further, leakage from sewage systems could also contaminate surface water (Parveen et al. 2008). It is possible that bats could acquire RVA infection while drinking water from contaminated water bodies. We previously identified *Salmonella sp.* in *P. medius* in Bangladesh and hypothesized that bat may have been infected via consuming contaminated water in close proximity to people and livestock (Islam et al. 2013).

The G1 strain of RVA that we detected in bats has primarily been isolated from people, both globally and in Bangladesh (Ahmed et al. 2010; Saudy et al. 2017). This genotype has also been detected in environmental water samples and bivalve shellfish samples (Kittigul et al. 2015), which supports our hypothesis that human RVA strains may contaminate local water sources. G1 has rarely been detected in animal species worldwide (Do et al. 2016), though it has previously been identified in cattle of India (Kumar and Malik 2018). This G1 strain has not previously been identified in a bat species, and though it is phylogenetically distinct from other G1 strains in people, they are 98–99% identical.

G8 genotypes are commonly detected in people in Africa and rarely detected elsewhere in the world (Agbemabiese et al. 2015). This genotype was also detected in people in Hungary within a zoonotic strain of RVA that spread from sheep and goats to people (Marton et al. 2017). G8 has also previously been identified from several livestock species (Karayel et al. 2017; Alkan et al. 2012; Gouvea et al. 1994), roe deer (Jamnikar-Ciglenecki et al. 2017) and poultry (Beserra et al. 2014). The G8 genotype identified in this study is closely related to RVA strains detected in people, bovine and caprine species. It is also possible that transmission of the RVA from livestock to bat also occurs, as there is evidence for these bats being infected with bovine and avian coronaviruses (Anthony et al. 2013). Given the described habitat and water-sharing behavior among bats, humans and livestock, it is not surprising to identify this G8 genotype in bats. A previous study provided evidence of direct bovine to human transmission of the RVA G8 genotype (Komoto et al. 2016). Human-associated bocaviruses and coronaviruses have been previously identified in *P. medius* in Bangladesh (Anthony et al. 2013). Together, these results suggest the possibility of host switching or virus spillover of RVA from people to bats. But we are not removing the possibility of shedding of bats’ own RVA in guano. So it needs further study to explore whether the RVAs detected in this study are their own virus or from an external source.

RVA is not known to be pathogenic to bats, and the ability to genotype RVA from bats may have been unsuccessful if the bats have a relatively low viral load, or only shed RVA in the feces at low levels. There were several limitations to our study. We only sampled three species of bats and had relatively small sample sizes for each species. The primers and probes that we used were not specifically designed to identify bat-associated RVA, but for identifying strains of RVA commonly found in human or environmental samples. Future studies should use assays designed to detect conserved regions of the RVA genome to avoid false positive and negative results. Future studies should be done using bat-specific primers or next-generation sequencing (NGS) followed by sequencing of other gene segments like VP6, NSP4, NSP1 and NSP5.

This study identifies a potentially significant human pathogen (RVA) in bats. This suggests that anthropozoonotic transmission may be occurring, which we hypothesize occurs via contaminated water sources. This study recommends that bats should be included when seeking to identify the pool of RVA strains that may reassert before spilling back over into humans.
